# The Impact of Water and Road Salt with Anti-Caking Agent on the Stiffness of Select Mixes Used for the Road Surface

**DOI:** 10.3390/ma14061345

**Published:** 2021-03-10

**Authors:** Piotr Mackiewicz, Eryk Mączka

**Affiliations:** Faculty of Civil Engineering, Wrocław University of Science and Technology, 50-370 Wrocław, Poland; eryk.maczka@pwr.edu.pl

**Keywords:** brine, sodium chloride, stiffness modulus, X-ray

## Abstract

An original experimental method was used to investigate the influence of water and road salt with anti-caking agent on the material used in pavement construction layers. This method allowed for monitoring material changes resulting from the influence of water and road salt with anti-caking agent over time. The experiment used five different mineral road mixes, which were soaked separately in water and brine for two time intervals (2 days and 21 days). Then, each sample of the mix was subjected to tests of the complex module using the four-point bending (4PB-PR) method. The increase in mass of the soaked samples and the change in value of the stiffness modulus were analyzed. Exemplary tomographic (X-ray) imaging was performed to confirm the reaction of the road salt and anti-caking agent (lead agent) with the material. Based on measurements of the stiffness modulus and absorption, the correlations of the mass change and the value of the stiffness modulus were determined, which may be useful in estimating the sensitivity of mixes to the use of winter maintenance agents—e.g., road salt with anti-caking agent (sodium chloride). It was found that the greatest changes occur for mixes intended for base course layers (mineral cement mix with foamed asphalt (MCAS) and mineral-cement-emulsion mixes (MCE)) and that the smallest changes occur for mixes containing highly modified asphalt (HIMA).

## 1. Introduction

In winter, the pavement layers are exposed to various environmental factors: heavy rainfall, snowfall, and rapid temperature change (changing the state of rainwater). Unfavorable conditions mobilize road services to ensure the safety of traffic on the road by the use of various chemical agents, in particular, road salt with anti-caking agent (sodium chloride (NaCl)). Road salt with anti-caking agent is an agent commonly used to remove snow and ice from roads [1]. The first successful use of salt for de-icing the surface course took place in the USA on New Hampshire roads in the winter of 1938. Since then, sodium chloride has been widely used in Northeast Europe and North America [2]. Salt use has increased significantly since its first use, e.g., to around 20 million tons of salt per year on roads in North America [3,4,5].

Brine for winter road maintenance should have a concentration of 20–25%, while the moistened pavement salt should contain 30% brine (NaCl or CaCl_2_ solution) with a concentration of 20–25% and 70% dry NaCl salt [6]. This form is characterized by rapid and effective melting of ice and snow to a temperature of ‒9 °C [7]. In many European countries, including Poland, road salt with anti-caking agent is commonly used, which is a mix of sodium chloride (96% NaCl), calcium chloride (2.5% CaCl_2_), and 0.2% anti-caking agent (potassium ferrocyanide (C_6_FeK_4_N_6_)). The additive prevents the formation of lumps of salt due to improper storage conditions or excessive exposure to moisture and low hygroscopic properties of the salt.

The influence of salt, although ensuring safety conditions on the pavement, significantly affects the strength properties of pavement layers. This applies not only to the upper surface of the surface course but also to the deeper layers. Rainwater as well as brine solution, which is mixed with snow or water, reaches the surface and within the road in various ways:

1. Through surface damage, e.g., fatigue cracks resulting from operation (mainly top-down)—the mix moves by gravity and is pressed under pressure from vehicle wheels to the inside of the crack.
2. During reconstruction of the pavement in winter, when vehicle traffic is allowed on a milled surface course. The solution penetrates directly into the upper part of the bonding layers or the substructure as a result of pressure and friction from vehicle wheels.
3. As a result of the inter-layer leakage and poor shape conditions of the ground shoulder.
4. As a result of aerosol dispersion of saline water droplets in the air by passing vehicles at high speed and settling on other areas of the road, including those that were not covered with salt.

It is worth mentioning that the action of water and salt, influencing the change in material strength and material properties, also leads to crucial changes in fatigue life. Salt is hygroscopic and attracts water. In winter maintenance, the surface is filled with water saturated with salt. Salt asphalt concrete can hold up to 10 percent extra water, which will expand upon subsequent freezing, causing additional destruction. In addition, it may have a negative impact on the environment itself (effects of soil acidification in the vicinity of the road lane) and cause corrosion of bridges, vehicles, road infrastructure elements, and roadside green. Despite the fact that the impacts are seasonal, it is assumed that excess and accumulation of chloride ions in a cyclical manner accelerates degradation of the material through maintenance.

Freezing and thawing cycles admittedly are a key factor when it comes to damage to the asphalt pavement in winter. Temperature fluctuations cause the moisture to constantly freeze and melt. When temperatures are above freezing, melting snow or rainwater seep into small cracks in the pavement. When re-freezing, it expands and cracks the pavement. The impact of road salt (mechanisms and chemical reaction between salt and material) and its influence on the pavement are probably the second key factors; therefore, it cannot be skipped. It is worth highlighting the sensitivity of the impact: the greater the impact, the worse the condition of the pavement (various other damages) and the traffic intensity.

## 2. State of Knowledge Review

The first studies related to the applicability of these maintenance measures for road surfaces date back to the late 1980s [8]. Susan et al., in their work, tried to develop a mathematical model describing defrosting of an ice-covered surface course depending on the location of the agent used, temperature, and its amount. On the basis of the analyses, the authors indicated that sodium chloride is a cheap and effective means of defrosting large areas in a short time in relation to the comparable calcium chloride. For several years since 1987, the simultaneous influence of water and road salt (sodium chloride) on road mixes has not been dealt with. The widespread availability and action of salt and the failure to register its negative effects resulted in little interest in this subject. It was only as a result of severe winters that the attention was refocused on measures supporting the improvement of road traffic safety in periods of heavy snowfall and frost. In 2002, Hassan et al. [9] published an article in which, based on the indirect tensile test, various freeze–thaw cycles, and the share of de-icing agents, they indicated that greater damage to bituminous materials (both in aggregate and binder) is a result of the action of various road salts and other agents compared to the action of water alone. 

In 2004, Kosior et al. [10] analyzed concrete and concrete with the addition of an asphalt binder used in road pavements in terms of material resistance to the applied freeze–thaw cycles with the use of water and, separately, a road salt solution with a concentration of 3%. The research showed that concrete with the addition of an asphalt binder better withstands the destructive activity of salt expressed by mass loss from 16 to 18 times depending on the cement used.

In the work of [11] in 2008, the authors analyzed various concrete mixes that were distinguished by different road salt content. It was suggested that, on the basis of the imaging performed, it can be presumed that chlorides affect the properties of the concrete mix. A prototype technique was used to detect the presence of chlorides and water in the layers of the pavement structure, based on wave measurements. 

In 2013, Serin et al. [12] published interesting work on the resistance of bitumen mixes to water and frost without the presence of salt in very cold regions. Attempts have been made to reflect the in situ conditions by using a different number of freeze–thaw cycles. Using ultrasound methods, scientists found a significant impact of the change in the state of water concentration on the tested mineral–asphalt mixes, for which it has been estimated that the damage to the material can reach up to 35%.

In 2014, Amini et al. analyzed the mass change of fine-grained bitumen mixtures, taking into account freeze–thaw cycles for an aqueous solution and the presence of various chemicals (including road salt) [13]. Based on mass changes and Marshall’s research, it was indicated that the tested bituminous mixtures are very susceptible to water and salt—they lose from 17 to 52% of their original strength and up to 22% of their original mass. In the same year, Liu et al. [14] published a paper that analyzed the effect of the brine of various concentrations (e.g., 3%, 5%, 10%, and 15%) on the stiffness determined in a static three-point bending test. It has been shown that the use of sodium chloride lowers the freezing point of water and that chlorides have an unfavorable effect on asphalt concrete, reducing its strength by up to 50%.

In [15], the authors analyzed the solubility of salt depending on the temperature. It was found that the solubility stabilizes at 0 °C at the concentration level of 35.8%.

In 2016, Hossain et al. [16] measured the thermal condition of various pavements in winter depending on the type of de-icing agent used (including salt) and different amounts of snow. Measurements have shown that the salt’s effectiveness is greater on asphalt concretes than on ordinary concretes. Tsang et al. [17] presented research on porous asphalt concrete, in which they analyzed various combinations of freeze–thaw cycles with the participation of water and the salts melted in it: calcium chloride II, magnesium II chloride, and sodium chloride I—road salt. It has been proven that sodium chloride has the greatest impact on mass change, and its loss after freeze–thaw cycles can reach up to 41%. It was indicated that the effects of de-icing agents were not checked in the time perspective, which is the desired issue.

The results of the experimental method studying the effect of salt (calcium chloride and its hydrates) on unpaved roads—breakstone and soil surfaces—were developed in 2019 by Almasi et al. [18]. They showed that the use of calcium chloride reduces soil moisture while reducing the value of the plasticity index by up to 25%. The effect increased caking of the ground layers and lowered load capacity.

Saltanovsa et al. [19] analyzed the effect of road salt on the efficiency of the method analyzing changes in the electromagnetic field, which allows for determining the condition of bituminous pavements (damage monitoring). The experiment in laboratory and in situ conditions showed that the presence of road salt significantly disrupts the efficiency of the presented method and decreases its effectiveness. For 1% brine concentration, the measurement error is 20%, which makes the developed method unsuitable for use.

In 2020, a key work by Gandara et al. [20] was published, in which the authors focused on the influence of road salt on the properties and parameters of 3 types of bituminous mix combinations (PA 16—porous asphalt on 50/70 ordinary asphalt and AC16 asphalt concrete): immersed in a salt solution, as an additive to aggregate and aggregate additive and soaking in salt. Various salt concentrations of 3.5%, 5%, and 10% were taken into account, the soaking time was 3 days, and the test temperature was 20 °C. The authors showed that mixes treated with salt as a result of soaking reduce their initial stiffness modulus (about 6%), but the changes are not as significant as when salted aggregate was applied to the composition of the mixture and then soaking was implemented (decrease by 70%). The fatigue life was also assessed, which for samples made of salted aggregate and soaked in brine decreased by an average of 50%.

A similar topic was analyzed by Zamanillo et al. [21]. The effect of brine with a percentage concentration of 5% at a temperature of 20 °C was investigated during the 2-day test on the bitumen AC 16 subjected to soaking and freeze–thaw cycles. The Indirect Tension Strength (ITS) method was used to test the stiffness, and the four-point bending (4PB-PR) test was used to determine the fatigue life. Moreover, the water and frost resistance test procedure (ITSR) was used. The research shows that the ITSR index for samples soaked in salt with samples soaked in water decreased by 1%, that the stiffness modulus decreased by 5%, and that the fatigue life decreased by about 30%.

The literature review shows that the topic of the influence of salt on the mixtures used in pavement construction layers is and should be constantly analyzed, if only due to various new road materials and complex conditions of impact. However, current studies mostly focus on the study of water and frost activities (freeze–thaw cycles) and their mutual correlation. There is a lack of water and de-icing agents’ analysis, which refers to, e.g., rain influence, or humid or chemical aggression. The vast majority of items mentioned define the results of tests carried out on typical asphalt mixtures. The conditions for the impact of de-icing agents and various levels of brine concentrations (especially those that may occur in the winter after intensive salting of the pavement, e.g., 20%) were not analyzed. No correlation was sought for their influence on the mechanical parameters of the mixtures—the stiffness modulus determined, e.g., by the 4PB-PR method (which reflects well the behavior of pavement layers under load). Different combinations of temperature soaking and solution flow simulation were also not used. 

The aim of this article is to show, on the basis of experimental studies, the sensitivity of the action of water and road salt to the stiffness of various asphalt mixtures in the long term. Environmental variables simulating in situ conditions were used. In the evaluation of material changes in mixes, the change in sample mass and the change in the stiffness modulus were analyzed. Using the X-ray tomographic method, the extent of material destruction is shown.

## 3. Materials and Methods

Three specific type of mixes for top asphalt pavement layers and two mixes for base coarse using recycle asphalt milling and, additionally, an innovative hydraulic binder were prepared for the experiment. This binder consisted of 40% CEM I 32.5R cement, 20% hydrated lime (Ca(OH)_2_), and mainly 40% dusty cement byproducts. Mixes containing this binder have been optimized for composition and material properties in previous research work [22,23,24,25]. Table 1 presents the detailed compositions of the analyzed mixes.

Mixes formed for prismatic beams with dimensions of 60 mm × 50 mm × 380 mm were placed in a closed container with a maximum capacity of 130 L. Inside the container, on its walls, was placed four independent pumps resistant to road salt with an anti-caking agent with an average pumping capacity of 7500 L/h, forcing steady circulation of the solution. The introduction of these devices was aimed at two aspects:

1. The brine concentration must be constant and well-distributed everywhere in the container (no local concentration points).
2. Forced circulation causes cyclical pressure of the solution on the material—an approximation of in situ conditions, in which the vehicle wheel “forces” the mixture under pressure into the road material.

**Figure 1:**
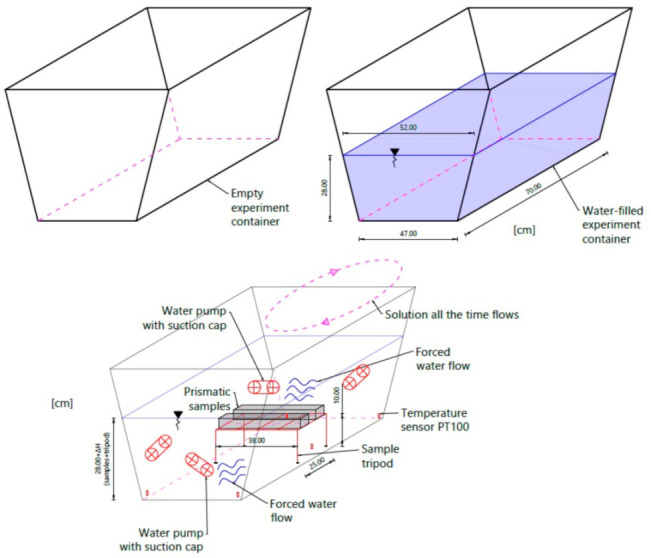
Experiment idea diagram.

**Figure 2:**
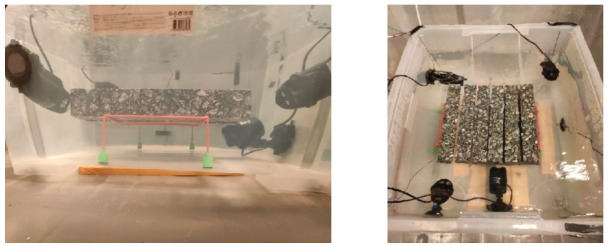
Fully armed container prepared to experiment.

Water- and salt-resistant sensors for temperature measurement were installed at five locations in the container near the bottom, which allowed us to track the changes in temperature during the test and when a fixed amount of road salt with an anti-caking agent was added to the water. The entire set was placed in the thermal chamber. The container was filled with water in such a way to know its quantity, density at the test temperature, and that the soaked samples were immersed to a depth of about 18 cm. The scheme of the test stand at the stage of soaking the samples is shown in Figure 1 and Figure 2.

**Figure 3:**
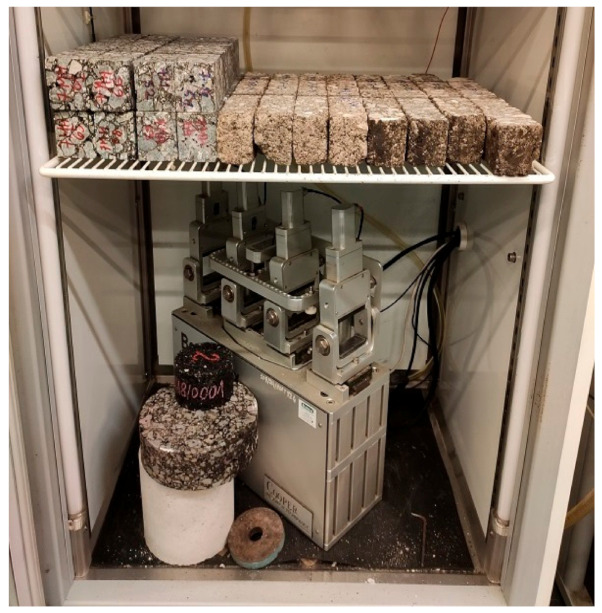
Testing machine and first sample series.

Of the nine specimens prepared for this study (for each type of mix), four were assigned to soaking in water and five were assigned to soaking in brine (target brine percentage concentration of the saline solution was 20%). The temperature at which the samples were soaked was 10.0 ± 0.2 °C. Samples of all mixes were accurately weighed prior to soaking. Then, each sample of a given mixture was subjected to tests of the complex module using the 4PB-PR method according to [26] at a temperature of 10.0 °C. The mixes were then soaked at two-time intervals (2 days and 21 days). The changes in mass and the stiffness modulus were analyzed. The mass measurements of the samples were performed according to the procedure described in [27]. Figure 3 shows the test stand for testing the stiffness modulus.

The principles adopted in the research method were established on the basis of phenomenon occurring in the winter and basic technological practices used in Poland. The equivalent temperature of 10 °C was adopted for the design of pavement layers using catalogue methods, e.g., [28,29], and was used in the stiffness test of most mixes [26]. The applied percentage of 20% brine was a compromise between saturated and unsaturated solutions. In fact, in winter, there is never a situation where we get saturation or supersaturation of the solution. This would be the case with a thin layer of snow/ice and too frequent road salt with an anti-caking agent. The task of water pumps with a flow of 7500 L/h was to simulate the interaction of variable snow/ice–salt environmental conditions related to the interaction of vehicle wheels.

The last part of the research was to visualize the behavior of the material after soaking in brine on one selected mix and to show the salt concentration on the surface of the material. For this purpose, observations were made in computed tomography using X-ray radiation. 

## 4. Results

### 4.1. Preliminary Research

In the field of preliminary examination (taking into account the variability of the concentration of the salt solution on the pavement), the change in water temperature with the change in salt concentration by 10% and 20% was analyzed.

Sample masses (*m_s_*, g) were calculated on the basis of the solution concentration (*C_p_*, %) and the mass of water (*m_w_*, g) according to dependency (1):

(1) $$m_{s}=\frac{C_{p}\cdot m_{w}}{100\%-C_{p}}\;\left[g\right]$$

**Figure 4:**
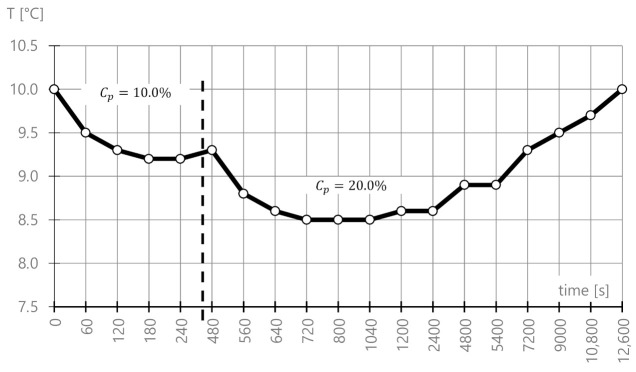
Temperature variability via adding road salt.

The calculations and measured values are shown in Table 2, while the graph of monitored values is shown in Figure 4.

The measurements show that, at the peak moment of saturation (for *Cp* = 10%), the temperature dropped by about 1.5 °C. For an increased concentration of 20%, temperature stability took place for approximately 12,600 s. Thanks to these preliminary examinations, the period needed to soak the samples in brine in order to obtain the thermal state of equilibrium and to maintain the consistency of the results of all tested mixes.

It should be mentioned that each of the prepared mixtures was soaked first in water only and then in the brine solution. This procedure was to simulate the initial impact of wet snow/ice, and then the conditions after the salt was spilt on the pavement.

### 4.2. Results for AC11S

**Figure 5:**
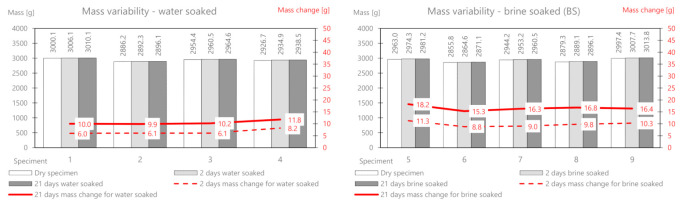
AC11S mix mass variability.

First, the AC11S mix containing 50/70 plain soft asphalt was analyzed. Before soaking, all samples were weighed and their initial stiffness was determined, and then the selected samples were soaked in water and brine. The results of the mass measurement related to absorption of the solution and the relative changes are presented in Table 3. Figure 5 shows the comprehensive results of the mass change for AC11S.

**Figure 6:**
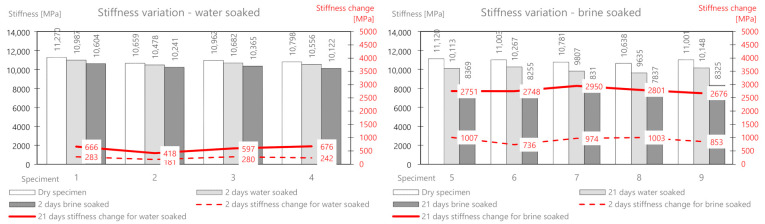
AC11S mix stiffness variation via water and brine soaking.

On the basis of the performed measurements, it should be concluded that the tested samples of the mixture are homogeneous—the dry mass variation index is less than 2%. Water absorption in the samples after two days of soaking is on average 6.6 g, while in brine, it is 9.84 g. The difference between the mass soaked in water and the mass soaked in brine (after 21 days) is 6.12 g. This is the average amount of salt deposited on the surface and in deeper areas of the samples. It should be noted that the salt enters the pores and reacts with the material, affecting its stiffness modulus. In a further part of this work, the change in the stiffness modulus for samples in a dry state after soaking in water and brine at two-time intervals (2 and 21 days) was analyzed. The results are presented in Table 4 and Figure 6.

The tests of the dry batch showed that the samples had similar values of the stiffness modulus, on average 10,915 MPa. After soaking the samples in water, a slight decrease in the modulus was observed both after 2 days and 21 days. Ultimately, the stiffness modulus changed by an average of about 590 MPa after the full test period (which is about 5.4% of the initial value). The brine soaking compared to the water treatment showed a clear and significant change in the initial stiffness modulus. Its value dropped by 915 MPa (about 8.4%) after 2 days, and after 21 days it dropped by as much as 2785 MPa (about 26%) compared to the initial value.

### 4.3. Results for AC WMS 22 (Pmb 25/55-60)

Another tested mix was the high modulus AC 22 WMS mix made on the common modified asphalt Pmb 25/55-60. The material is commonly used for the bonding layer and for the framework subjected to heavy and very heavy movement. 

**Figure 7:**
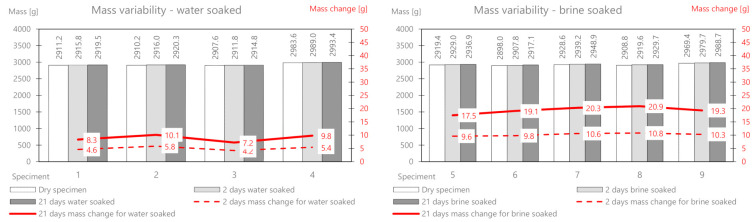
ACWMS22(Pmb) mix mass variability.

As part of the experiment, nine prismatic specimens were also prepared. Then, the research material was selected—a part (four bars) was intended for soaking in water, and a part (five bars) was intended for brine at a fixed concentration. The developed results related to mass changes as a result of the interaction between water and salt are presented in Table 5 and presented in Figure 7.

**Figure 8:**
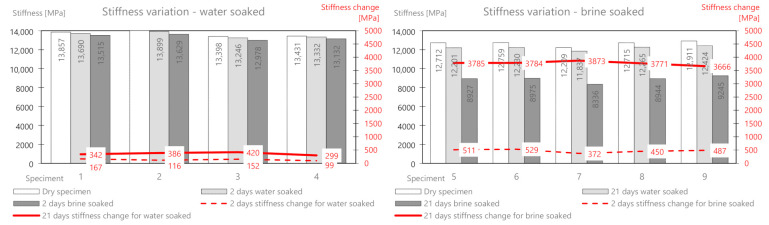
ACWMS22(Pmb) mix stiffness variation via water and brine soaking.

The mass change as a result of water absorption after two days of soaking is on average 5.0 g and that for brine is 10.22 g. The mass of the deposited salt after 2 days is 5.22 g, and after 21 days, it is 10.57 g. The change in the stiffness modulus is presented in Table 6 and Figure 8.

As for the previous mix, after soaking in water only, a slight decrease in the stiffness modulus was observed both after 2 days (134 MPa) and 21 days (362 MPa). The mix, therefore, appears to be water-resistant. The brine-soaked treatment compared to the water treatment showed a clear and significant change in the stiffness modulus. Its value decreased by 470 MPa (about 3.7%) after 2 days, and after 21 days, it decreased by as much as 3776 MPa (about 30%) compared to the initial value.

### 4.4. Results for ACWMS 22 (Pmb 25/55-80 HIMA)

**Figure 9:**
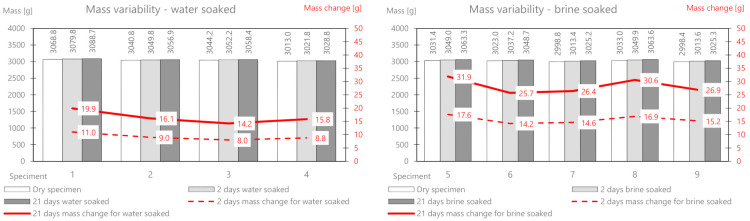
ACWMS22(HIMA) mix mass variability.

The AC 22 WMS mix was made of highly modified asphalt Pmb 25/55-80 highly modified asphalt (HIMA), which is characterized by both high fatigue strength and resistance to permanent deformation. The composition and raw material of the ACWMS 22 (HIMA) mineral mix are identical compared to the previously discussed ACWMS 22 (Pmb) mix. The only difference is the type of asphalt binder used. The developed results related to absorption of the solution after 2 and 21 days are presented in Table 7 and Figure 9.

**Figure 10:**
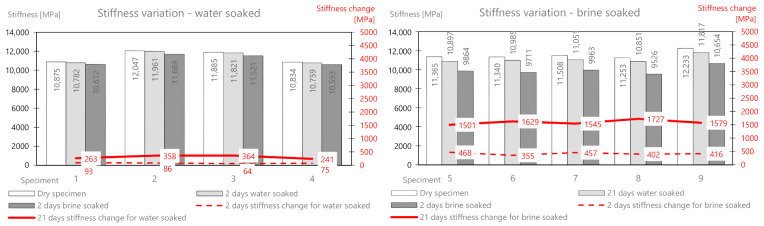
ACWMS22(HIMA) mix stiffness variation via water and brine soaking.

The mass change as a result of water absorption after two days of soaking is on average 9.2 g, and that for brine is 15.7 g. The mass of the deposited salt after 2 days is 6.5 g, and after 21 days, it is 11.8 g. This is a greater value than for the AC11S and ACWMS 22 mix (Pmb). The change in the stiffness modulus is presented in Table 8 and Figure 10.

After soaking in water only, a slight decrease in stiffness modulus was also observed after both 2 days (80 MPa) and 21 days (307 MPa). Brine treatment compared to water treatment again showed a clear and significant change in the stiffness modulus. Its value dropped by 420 MPa (about 3.6%) after 2 days, and after 21 days, it dropped by 1596 MPa (about 14%) compared to the initial value.

### 4.5. Results for MCAS

**Figure 11:**
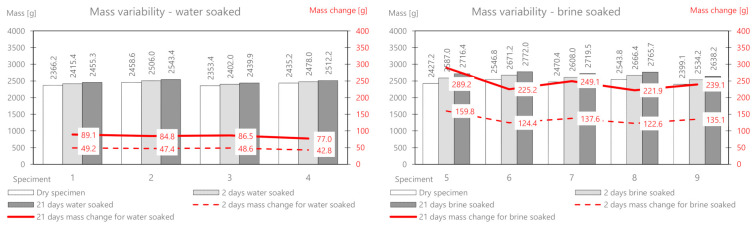
MCAS mix mass variability.

The mineral cement mix with foamed asphalt (MCAS) mixture, intended mainly for base course layers, showed large changes in the mass of samples soaked in water and brine (Table 9 and Figure 11).

**Figure 12:**
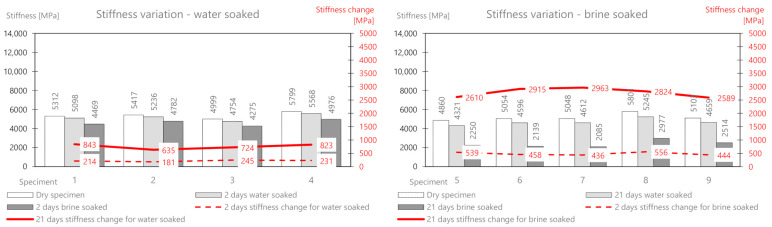
MCAS mix stiffness variation via water and brine soaking.

The mass change as a result of water absorption after two days of soaking is on average 47 g, and that for brine is 135.9 g. The mass of the deposited salt after 2 days is 88.9 g, and after 21 days, it is 160.54 g. There is a significant influence from the different structures of this mix related to greater porosity. In the following part, the change in the stiffness modulus is analyzed. The results are presented in Table 10 and Figure 12.

After soaking in water alone, a greater decrease (than for the previous mixes) of the modulus is also observed after both 2 days (218 MPa) and 21 days (756 MPa). The brine treatment compared to the water treatment again showed a clear and significant change in the stiffness modulus. Its value dropped by 487 MPa (about 9.4%) after 2 days, and after 21 days, it dropped by 2780 MPa (about 54%) compared to the initial value.

### 4.6. Results for MCE

**Figure 13:**
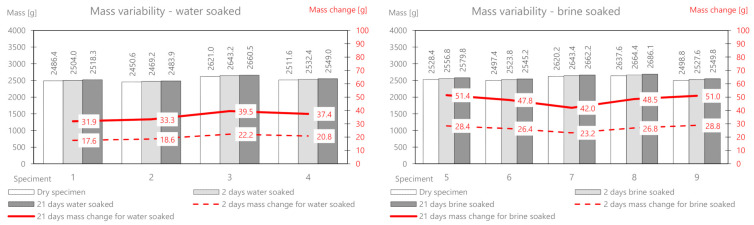
MCE mix mass variability.

The mineral-cement-emulsion (MCE) mix is also intended for base course layers and shows large changes in the mass of the samples soaked in water and brine (Table 11 and Figure 13).

The mass change due to the absorption of water after two days of soaking is on average 20 g, and that for brine is 26.72 g (about half as much as for MCAS). The mass of the deposited salt after 2 days is 6.92 g, and after 21 days, it is 12.6 g. A slightly smaller influence of the less porous structure of the mix is visible.

**Figure 14:**
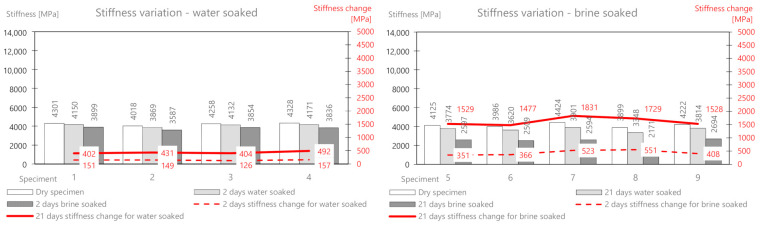
MCE mix stiffness variation via water and brine soaking.

The change in the stiffness modulus was further analyzed. The results are presented in Table 12 and Figure 14.

After soaking in water alone, about half the decrease (compared to MCAS) in the module was observed after both 2 days (146 MPa) and 21 days (432 MPa). Brine soaked maintenance, compared to water, showed a change in the stiffness modulus by 440 MPa (about 11%) after 2 days, and after 21 days, it decreased by 1619 MPa (about 39%) compared to the initial value.

It can therefore be concluded that road salt with an anti-caking agent significantly weakened the tested mixes by lowering the stiffness modulus. This observation proves that the action of water and brine leads to uniform destruction of the material. It depends on the composition of the mixtures and their structure. Later in this article, the results for all tested mixtures were collected and compared.

### 4.7. Tomography Imaging Salt in a Selected Mix

The results of the research, and in particular the mass changes, confirm that salt has a destructive effect and penetrates the structure of the mixes. As part of a further part of the analysis, imaging using computed tomography was performed to analyze the depth at which salt is deposited and how it reacts with it while reducing its strength parameters. 

**Figure 15:**
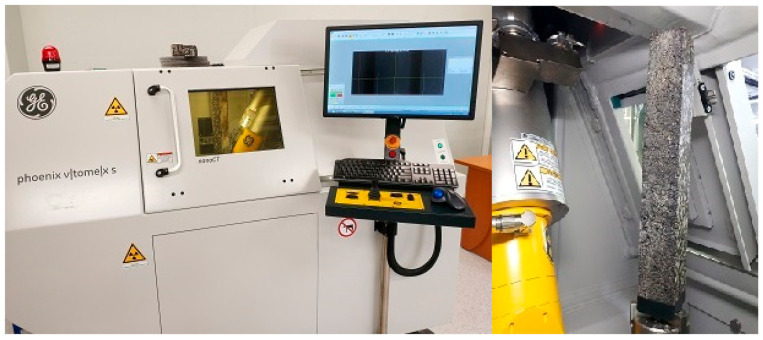
Computed tomography and mounted specimen.

**Figure 16:**
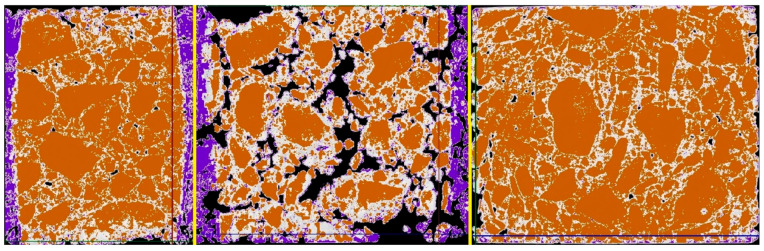
Edge specimen view.

**Figure 17:**
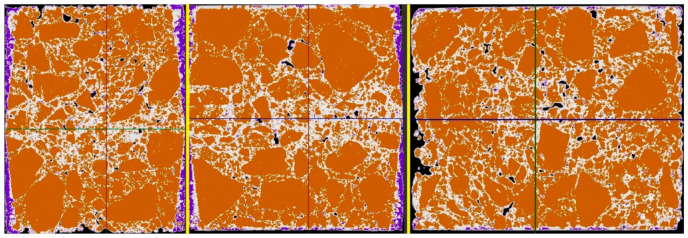
Middle specimen view.

Computed tomography scanning was performed on a sample of the AC11S mix that was soaked in saline for 21 days. After data preparation and data acquisition, two key shots of the x-rayed material were taken—shown in 3 planes at the very edge of the material and shown in 3 planes passing through the center of the material. Analyzing the results, 4 color ranges were selected, which directly define the variable structure of the x-rayed material. Orange is the color of the mineral mix, white is the asphalt binder, and purple is the material degraded by road salt with anti-caking agent. Black means air voids. The test machine used together with the sample is presented in Figure 15, while the views discussed are presented in Figure 16 and Figure 17, which show, respectively, the side, front, and top sections of the exposed sample.

The main salt clusters are on the surface of the X-rayed element at a maximum depth of 2.7 mm. The volumetric analysis with the use of tomographic imaging showed that, for the AC11S mix, about 5.51% of the material was degraded by the effect of salt. It is worth recalling that, for this mix, after 21 days of soaking in brine, about 6.12 g of salt deposited on the surface and in deeper areas of the sample. At a later stage of this work, the authors will conduct research for other mixtures in order to determine the correlation between mass changes and the volume of material destruction.

## 5. Discussion

In terms of the impact of changes in the interaction of road salt with anti-caking agent and water, five different road mixes used in the pavement were analyzed. 

High Modulus Asphalt Concrete (HMAC) mixes were used in the article contain polymer modified asphalt—Pmb 25/55-60 and 25/55-80 (HIMA). Due to modification in the asphalt matrix, which contains different amounts of SBS polymer, the required stiffness modulus for typical HMAC mixes (with common asphalt like 20/30) used in the binder course—14,000 MPa—or base course—11,000 MPa—is difficult to reach or to be attained. It is caused by the internal properties of the binder that lead the material to being more flexible. Nowadays, it is a general problem for producers, distributors, and scientists because the admission of Pmb and HIMA binder’s usage into HMAC mixes creates a problem related to the required value of stiffness modulus in some regulations [30]. In this article, the stiffness modulus value for AC22WMS with common Pmb 25/55-60 samples reaches or is close to 14,000 MPa and that for AC22WMS with HIMA asphalt (25/55-80) is close to 12,000 MPa.

In this order (before the mixtures were tested), it was examined how road salt affected the temperature change. Water as a liquid is a set of water molecules that are mutually arranged in relation to each other and are connected with each other by hydrogen bonds. A chemical reaction takes place when salt is added to the water. The hydrogen bonds are broken, and the water molecule is arranged with appropriate charges against the dissolved sodium Na+ cation and the Cl− chloride anion. The observations from the experiment confirm (Figure 4) that, after adding road salt (e.g., sprinkling an iced surface with salt), the system needs external energy for the entire dissolution process to take place—an endothermic reaction takes place (the reaction enthalpy changes). When heat is “taken” from the existing state—the temperature drops (for road materials, this situation introduces a risky shrinkage that activates the cracking process occurring as a result of energy changes in the system). The effect of lowering the temperature (using heat for the reaction) is to lower the freezing point of water. However, it should be remembered that the entire process is temporary—after dissolving the salt, the temperature of the system stabilizes.

**Figure 18:**
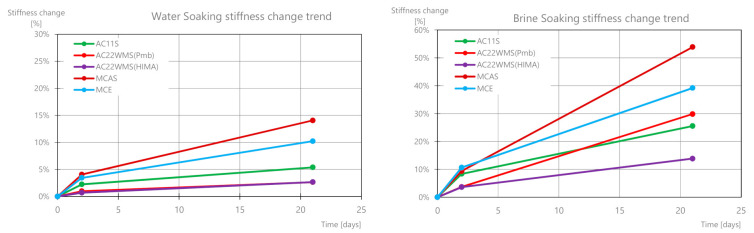
Results of the percentage changes in the stiffness modulus for different mixes over time.

High reproducibility of the results was found, obtaining similar results for samples of the same material. Figure 18 shows the percentage change in the value of the stiffness modulus for the tested mixtures under various conditions (soaking in water and salt after 2 and 21 days). It was found that the greatest changes occur for the base course mixtures: MCAS and MCE, which after 21 days reach 54% and 39%, respectively. The mixture of the high stiffness modulus with the highly modified asphalt AC22WMS (HIMA) showed the lowest sensitivity to salt. It should be noted that the effect of water itself also has a destructive effect on the mixtures and reduces the modulus value from 2% to 14%.

**Figure 19:**
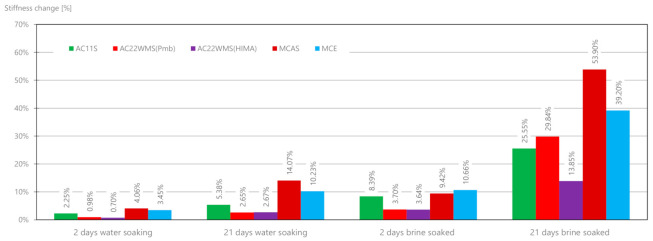
Stiffness modulus change results for various mixes.

Figure 19 shows the absolute stiffness modulus value changes. The largest change in the value of the module was found for the 21st day of brine soaking. It is about three to four times greater than for two days of soaking in water. The greatest change was achieved for the AC22WMS (Pmb), AC11S, and MCAS mixes.

By analyzing the absorption of water and brine for various mixes, it can be concluded that the mixes of MCAS and MCE show high absorption of water and salt solutions. Their mass changes are 9.9% and 1.9%, respectively. Although they are intended for the base course layers, in the absence of full adhesion of the layers and significant surface damage to the upper layers (cracks, potholes), they will not be resistant to salt treatments in winter maintenance. 

The other mixes are also characterized by a lower degree of absorption (up to 1%), but they constitute the upper layers of the pavement and the degradation process for them will be equally intense and dangerous.

**Figure 20:**
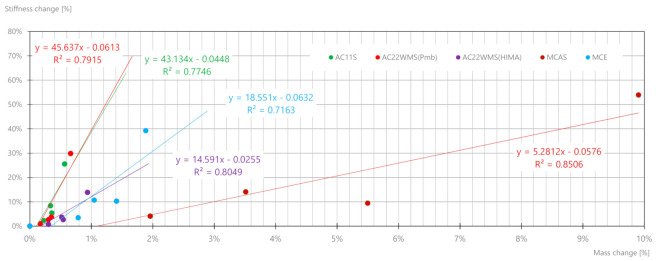
Correlations between modulus change and mass change as a result of the soaking phenomenon for various mixes.

The effect of water and salt on mixtures is reflected in the reduction in the value of the stiffness modulus; therefore, for soaked mixtures, correlations between modulus change and mass change as a result of soaking phenomenon were developed (Figure 20).

On the basis of the obtained dependencies, it is possible to estimate the change in the value of the stiffness modulus on the basis of the mass change associated with the absorption of the salt solution. It should be noted that mixes AC11S and AC22WMS (Pmb) have similar dependencies.

## 6. Conclusions

Our analyses showed that brine has a fundamental impact on changes in the mechanical properties of road materials. It causes an increase in mass and a change in the stiffness modulus. We found that a chemical reaction that occurs when road salt is added to water causes a temporary temperature fall. Probably the shrinkage during this reaction could accelerate material degradation (including aging) or could modify material rheological properties. However, it should be confirmed by other separate research.

The image recorded in tomography allows for indicating the extent of the impact of the salt solution and for determining the area of material degradation. The X-ray method can be effective in assessing the quality and, consequently, the durability of materials exposed to the effects of road salts. Changes in the structure are directly related to changes in the stiffness modulus. Salt depositing on the surface and reacting with the asphalt weakens the original material, which can cause reduced fatigue life.

We found that the greatest changes occurred for mixes intended for the base layers: MCAS and MCE. Modules changed after 21 days to 54% and 39%, respectively. Among the five tested mixes, the mix characterized by high stiffness modulus with highly modified asphalt AC22WMS (HIMA) showed the lowest sensitivity to salt. Therefore, it is necessary to recognize the properties of highly modified asphalt, thanks to which the WMS mix is able to withstand the salt solution well.

The determined correlations between mass change and changes in the value of the stiffness modulus may be useful in estimating the sensitivity of mixes to the application of winter maintenance treatments with road salt. Water with salt will penetrate the structure of mixtures intended for upper layers and substructures as well as in damaged and strongly cracked wear layers. It can cause a change in the value of modules and deterioration of durability. It is therefore important to pay attention to the tightness and state of damage of the pavement layers before using large amounts of salt in winter maintenance of road surfaces. For salt concentrations of 10–20%, significant changes in asphalt mixes and, consequently, in the pavement durability should be expected. Fatigue analyses will be the subject of further work by the authors. 

## Figures and Tables

**Table 1:** Road mixes used in experiment.

Mix	Bond Material
AC11S	Asphalt D50/70: 5.90% Amfibolit 8/11: 32.40% Amfibolit 5/8: 10.50% Sjenit 2/5: 13.80% Sjenit 0/2: 31.20% 0/1 milled stone extender: 6.20% Porosity: 3.70%
AC22WMS (Pmb)	Asphalt 25/55-60: 4.98% 16/22 gabbro grit: 28.50% 11/16 gabbro grit: 9.50% 8/11 gabbro grit: 8.55% 4/8 gabbro grit: 8.55% 2/5 gabbro grit: 15.20% 0/2 gabbro crushed sand: 17.10% 0/1 milled stone extender: 7.60% Porosity: 2.38%
AC22WMS (HIMA)	Asphalt 25/55-80 HIMA: 4.98% 16/22 gabbro grit: 28.50% 11/16 gabbro grit: 9.50% 8/11 gabbro grit: 8.55% 4/8 gabbro grit: 8.55% 2/5 gabbro grit: 15.20% 0/2 gabbro crushed sand: 17.10% 0/1 milled stone extender: 7.60% Porosity: 2.97%
MCAS	Asphalt destruct 0/10: 37.6% Natural ginning aggregate 0/2: 9.4% Crushed ginning aggregate 0/31.5: 47% Foamed asphalt 70/100: 3% Hydraulic binder: 3% (40% CEM cement I 32,5R, 20% hydrated lime (Ca(OH)2, 40% dusty cement byproducts) Porosity: 13.21%
MCE	Asphalt destruct 0/10: 34.4% Natural ginning aggregate 0/2: 8.6% Crushed ginning aggregate 0/31.5: 43% Asphalt emulsion 60/40: 5% Hydraulic binder: 3% (40% CEM cement I 32,5R, 20% hydrated lime (Ca(OH)2, 40% dusty cement byproducts) Water: 6% Porosity: 14.02%

**Table 2:** Brine data and measured temperature variability.

Brine					Temperature Shock	
Step	Temperature (°C)	Total Water Mass, *m_w_* (g)	Percentage Concentration (Target Value), *C_p_* (%)	Total Mass of Gritting Salt with Anti-caking Agent, *m_s_* (g)	Time (s)	Temperature Noticed (°C)
1	10.0	96,534.9	10.0%	10,726.1	0	10.0
					60	9.5
					120	9.3
					180	9.2
					240	9.2
2	10.0	96,534.9	20.0%	24,133.7	480	9.3
					560	8.8
					640	8.6
					720	8.5
					800	8.5
					1040	8.5
					1200	8.6
					2400	8.6
					4800	8.9
					5400	8.9
					7200	9.3
					9000	9.5
					10,800	9.7
					12,600	10.0

**Table 3:** Mass variability of AC11S via soaking in water and brine.

Specimen Index	Mass					Mass Change			
	Dry Specimen	Water Soaked		Brine Soaked		Water Soaked		Brine Soaked	
	Initial State	2 Days	21 Days	2 Days	21 Days	2 Days	21 Days	2 Days	21 Days
	(g)	(g)	(g)	(g)	(g)	(g)	(g)	(g)	(g)
1	3000.1	3006.1	3010.1			6.0	10.0		
2	2886.2	2892.3	2896.1			6.1	9.9		
3	2954.4	2960.5	2964.6			6.1	10.2		
4	2926.7	2934.9	2938.5			8.2	11.8		
5	2963.0			2974.3	2981.2			11.3	18.2
6	2855.8			2864.6	2871.1			8.8	15.3
7	2944.2			2953.2	2960.5			9.0	16.3
8	2879.3			2889.1	2896.1			9.8	16.8
9	2997.4			3007.7	3013.8			10.3	16.4
Average	2934.1	2948.5	2952.3	2937.8	2944.5	6.60	10.48	9.84	16.60
Standard deviation	48.5	41.2	41.4	53.3	53.2	0.9	0.8	0.9	0.9
The coefficient of variation (%)	1.65%	1.40%	1.40%	1.81%	1.81%	14.01%	7.38%	9.24%	5.66%

**Table 4:** AC11S mix stiffness variation via water and brine soaking.

Specimen Index	Stiffness					Stiffness Change			
	Dry Specimen	Water Soaked		Brine Soaked		Water Soaked		Brine Soaked	
	Initial State	2 Days	21 Days	2 Days	21 Days	2 Days	21 Days	2 Days	21 Days
	(MPa)	(MPa)	(MPa)	(MPa)	(MPa)	(MPa)	(MPa)	(MPa)	(MPa)
1	11,270	10,987		10,604		283	666		
2	10,659	10,478		10,241		181	418		
3	10,962	10,682		10,365		280	597		
4	10,798	10,556		10,122		242	676		
5	11,120		10,113		8369			1007	2751
6	11,003		10,267		8255			736	2748
7	10,781		9807		7831			974	2950
8	10,638		9635		7837			1003	2801
9	11,001		10,148		8325			853	2676
Average	10,914.7	10,675.8	9994.0	10,333.0	8123.4	246.5	589.3	914.6	2785.2
Standard deviation	199.8	193.9	235.1	178.5	239.1	41.1	103.4	105.4	91.5
The coefficient of variation (%)	1.83%	1.82%	2.35%	1.73%	2.94%	16.68%	17.56%	11.53%	3.29%

**Table 5:** Mass variability of AC 22 WMS(Pmb) via soaking in water and brine.

Specimen Index	Mass					Mass Change			
	Dry Specimen	Water Soaked		Brine Soaked		Water Soaked		Brine Soaked	
	Initial State	2 Days	21 Days	2 Days	21 Days	2 Days	21 Days	2 Days	21 Days
	(g)	(g)	(g)	(g)	(g)	(g)	(g)	(g)	(g)
1	2911.2	2915.8	2919.5			4.6	8.3		
2	2910.2	2916.0	2920.3			5.8	10.1		
3	2907.6	2911.8	2914.8			4.2	7.2		
4	2983.6	2989.0	2993.4			5.4	9.8		
5	2919.4			2929.0	2936.9			9.6	17.5
6	2898.0			2907.8	2917.1			9.8	19.1
7	2928.6			2939.2	2948.9			10.6	20.3
8	2908.8			2919.6	2929.7			10.8	20.9
9	2969.4			2979.7	2988.7			10.3	19.3
Average	2926.3	2933.2	2937.0	2935.1	2944.3	5.00	8.85	10.22	19.42
Standard deviation	28.2	32.3	32.6	24.6	24.5	0.6	1.2	0.5	1.2
The coefficient of variation (%)	0.96%	1.10%	1.11%	0.84%	0.83%	12.65%	13.24%	4.48%	5.99%

**Table 6:** AC22WMS(Pmb) mix stiffness variation via water and brine soaking.

Specimen Index	Stiffness					Stiffness Change			
	Dry Specimen	Water Soaked		Brine Soaked		Water Soaked		Brine Soaked	
	Initial State	2 Days	21 Days	2 Days	21 Days	2 Days	21 Days	2 Days	21 Days
	(MPa)	(MPa)	(MPa)	(MPa)	(MPa)	(MPa)	(MPa)	(MPa)	(MPa)
1	13,857	13,690		13,515		167	342		
2	14,015	13,899		13,629		116	386		
3	13,398	13,246		12,978		152	420		
4	13,431	13,332		13,132		99	299		
5	12,712		12,201		8927			511	3785
6	12,759		12,230		8975			529	3784
7	12,209		11,837		8336			372	3873
8	12,715		12,265		8944			450	3771
9	12,911		12,424		9245			487	3666
Average	13,111.9	13,541.8	12,191.4	13,313.5	8885.4	133.5	361.8	469.8	3775.8
Standard deviation	562.9	265.1	193.2	267.2	298.1	27.2	45.6	55.6	65.8
The coefficient of variation (%)	4.29%	1.96%	1.59%	2.01%	3.36%	20.38%	12.60%	11.83%	1.74%

**Table 7:** Mass variability of AC 22 WMS(HIMA) via soaking in water and brine.

Specimen Index	Mass					Mass Change			
	Dry Specimen	Water Soaked		Brine Soaked		Water Soaked		Brine Soaked	
	Initial State	2 Days	21 Days	2 Days	21 Days	2 Days	21 Days	2 Days	21 Days
	(g)	(g)	(g)	(g)	(g)	(g)	(g)	(g)	(g)
1	3068.8	3079.8	3088.7			11.0	19.9		
2	3040.8	3049.8	3056.9			9.0	16.1		
3	3044.2	3052.2	3058.4			8.0	14.2		
4	3013.0	3021.8	3028.8			8.8	15.8		
5	3031.4			3049.0	3063.3			17.6	31.9
6	3023.0			3037.2	3048.7			14.2	25.7
7	2998.8			3013.4	3025.2			14.6	26.4
8	3033.0			3049.9	3063.6			16.9	30.6
9	2998.4			3013.6	3025.3			15.2	26.9
Average	3027.9	3050.9	3058.2	3032.6	3045.2	9.20	16.53	15.70	28.30
Standard deviation	21.4	20.5	21.2	16.2	17.2	1.1	2.1	1.3	2.5
The coefficient of variation (%)	0.71%	0.67%	0.69%	0.54%	0.56%	12.01%	12.59%	8.43%	8.67%

**Table 8:** AC22WMS(HIMA) mix stiffness variation via water and brine soaking.

Specimen Index	Stiffness					Stiffness Change			
	Dry Specimen	Water Soaked		Brine Soaked		Water Soaked		Brine Soaked	
	Initial State	2 Days	21 Days	2 Days	21 Days	2 Days	21 Days	2 Days	21 Days
	(MPa)	(MPa)	(MPa)	(MPa)	(MPa)	(MPa)	(MPa)	(MPa)	(MPa)
1	10,875	10,782		10,612		93	263		
2	12,047	11,961		11,689		86	358		
3	11,885	11,821		11,521		64	364		
4	10,834	10,759		10,593		75	241		
5	11,365		10,897		9864			468	1501
6	11,340		10,985		9711			355	1629
7	11,508		11,051		9963			457	1545
8	11,253		10,851		9526			402	1727
9	12,233		11,817		10,654			416	1579
Average	11,482.2	11,330.8	11,120.2	11,103.8	9943.6	79.5	306.5	419.6	1596.2
Standard deviation	462.0	562.5	355.2	504.8	384.7	11.0	55.1	40.6	77.7
The coefficient of variation (%)	4.02%	4.96%	3.19%	4.55%	3.87%	13.85%	17.97%	9.67%	4.87%

**Table 9:** Mass variability of MCAS via soaking in water and brine.

Specimen Index	Mass					Mass Change			
	Dry Specimen	Water Soaked		Brine Soaked		Water Soaked		Brine Soaked	
	Initial State	2 Days	21 Days	2 Days	21 Days	2 Days	21 Days	2 Days	21 Days
	(g)	(g)	(g)	(g)	(g)	(g)	(g)	(g)	(g)
1	2366.2	2415.4	2455.3			49.2	89.1		
2	2458.6	2506.0	2543.4			47.4	84.8		
3	2353.4	2402.0	2439.9			48.6	86.5		
4	2435.2	2478.0	2512.2			42.8	77.0		
5	2427.2			2587.0	2716.4			159.8	289.2
6	2546.8			2671.2	2772.0			124.4	225.2
7	2470.4			2608.0	2719.5			137.6	249.1
8	2543.8			2666.4	2765.7			122.6	221.9
9	2399.1			2534.2	2638.2			135.1	239.1
Average	2444.5	2450.4	2487.7	2613.4	2722.4	47.00	84.36	135.90	244.90
Standard deviation	65.0	43.1	42.0	51.3	47.9	2.5	4.5	13.3	24.2
The coefficient of variation (%)	2.66%	1.76%	1.69%	1.96%	1.76%	5.34%	5.32%	9.78%	9.89%

**Table 10:** MCAS mix stiffness variation via water and brine soaking.

Specimen Index	Stiffness					Stiffness Change			
	Dry Specimen	Water Soaked		Brine Soaked		Water Soaked		Brine Soaked	
	Initial State	2 Days	21 Days	2 Days	21 Days	2 Days	21 Days	2 Days	21 Days
	(MPa)	(MPa)	(MPa)	(MPa)	(MPa)	(MPa)	(MPa)	(MPa)	(MPa)
1	5312	5098		4469		214	843		
2	5417	5236		4782		181	635		
3	4999	4754		4275		245	724		
4	5799	5568		4976		231	823		
5	4860		4321		2250			539	2610
6	5054		4596		2139			458	2915
7	5048		4612		2085			436	2963
8	5801		5245		2977			556	2824
9	5103		4659		2514			444	2589
Average	5265.9	5164.0	4686.6	4625.5	2393.0	217.8	756.3	486.6	2780.2
Standard deviation	325.0	291.9	303.3	271.4	327.3	23.9	83.3	50.5	154.3
The coefficient of variation (%)	6.17%	5.65%	6.47%	5.87%	13.68%	10.97%	11.01%	10.38%	5.55%

**Table 11:** Mass variability of MCE via soaking in water and brine.

Specimen Index	Mass					Mass Change			
	Dry Specimen	Water Soaked		Brine Soaked		Water Soaked		Brine Soaked	
	Initial State	2 Days	21 Days	2 Days	21 Days	2 Days	21 Days	2 Days	21 Days
	(g)	(g)	(g)	(g)	(g)	(g)	(g)	(g)	(g)
1	2486.4	2504.0	2518.3			17.6	31.9		
2	2450.6	2469.2	2483.9			18.6	33.3		
3	2621.0	2643.2	2660.5			22.2	39.5		
4	2511.6	2532.4	2549.0			20.8	37.4		
5	2528.4			2556.8	2579.8			28.4	51.4
6	2497.4			2523.8	2545.2			26.4	47.8
7	2620.2			2643.4	2662.2			23.2	42.0
8	2637.6			2664.4	2686.1			26.8	48.5
9	2498.8			2527.6	2549.8			28.8	51.0
Average	2539.1	2537.2	2552.9	2583.2	2604.6	19.80	35.53	26.72	48.13
Standard deviation	64.8	65.2	66.3	59.2	58.5	1.8	3.1	2.0	3.4
The coefficient of variation (%)	2.55%	2.57%	2.60%	2.29%	2.25%	9.12%	8.68%	7.42%	7.00%

**Table 12:** MCE mix stiffness variation via water and brine soaking.

Specimen Index	Stiffness					Stiffness Change			
	Dry Specimen	Water Soaked		Brine Soaked		Water Soaked		Brine Soaked	
	Initial State	2 Days	21 Days	2 Days	21 Days	2 Days	21 Days	2 Days	21 Days
	(MPa)	(MPa)	(MPa)	(MPa)	(MPa)	(MPa)	(MPa)	(MPa)	(MPa)
1	4301	4150		3899		151	402		
2	4018	3869		3587		149	431		
3	4258	4132		3854		126	404		
4	4328	4171		3836		157	492		
5	4125		3774		2597			351	1529
6	3986		3620		2509			366	1477
7	4424		3901		2594			523	1831
8	3899		3348		2171			551	1729
9	4222		3814		2694			408	1528
Average	4173.4	4080.5	3691.4	3794.0	2512.7	145.8	432.3	439.8	1618.5
Standard deviation	166.5	122.9	194.3	121.7	180.9	11.8	36.3	82.0	136.6
The coefficient of variation (%)	3.99%	3.01%	5.26%	3.21%	7.20%	8.08%	8.41%	18.65%	8.44%

## Data Availability

Not applicable.
